# Spatiotemporal Expression and Molecular Characterization of *miR-344b* and *miR-344c* in the Developing Mouse Brain

**DOI:** 10.1155/2016/1951250

**Published:** 2016-03-01

**Authors:** Jia-Wen Leong, Syahril Abdullah, King-Hwa Ling, Pike-See Cheah

**Affiliations:** ^1^Department of Human Anatomy, Faculty of Medicine and Health Sciences, Universiti Putra Malaysia (UPM), 43400 Serdang, Selangor, Malaysia; ^2^Genetics and Regenerative Medicine Research Centre (GRMRC), Faculty of Medicine and Health Sciences, Universiti Putra Malaysia (UPM), 43400 Serdang, Selangor, Malaysia; ^3^Clinical Genetics Unit, Department of Biomedical Sciences, Faculty of Medicine and Health Sciences, Universiti Putra Malaysia (UPM), 43400 Serdang, Selangor, Malaysia

## Abstract

MicroRNAs (miRNAs) are small noncoding RNA known to regulate brain development. The expression of two novel miRNAs, namely,* miR-344b* and* miR-344c*, was characterized during mouse brain developmental stages in this study.* In situ* hybridization analysis showed that* miR-344b* and* miR-344c* were expressed in the germinal layer during embryonic brain developmental stages. In contrast,* miR-344b* was not detectable in the adult brain while* miR-344c* was expressed exclusively in the adult olfactory bulb and cerebellar granular layer. Stem-loop RT-qPCR analysis of whole brain RNAs showed that expression of the* miR-344b* and* miR-344c* was increased as brain developed throughout the embryonic stage and maintained at adulthood. Further investigation showed that these miRNAs were expressed in adult organs, where* miR-344b* and* miR-344c* were highly expressed in pancreas and brain, respectively. Bioinformatics analysis suggested* miR-344b* and* miR-344c* targeted* Olig2* and* Otx2* mRNAs, respectively. However, luciferase experiments demonstrated that these miRNAs did not target* Olig2* and* Otx2* mRNAs. Further investigation on the locality of* miR-344b* and* miR-344c* showed that both miRNAs were localized in nuclei of immature neurons. In conclusion,* miR-344b* and* miR-344c* were expressed spatiotemporally during mouse brain developmental stages.

## 1. Introduction

In recent years, studies have shown that microRNAs (miRNAs) play a significant role in brain development. For instance,* miR-134* is localized to the synaptodendritic area in rat hippocampal neurons and is associated with synaptic development, maturation, and plasticity [1]. In mammals,* miR-9* is a neural-specific miRNA and was not found to be expressed in any other tissues.* miR-9 *is widely expressed in neural precursor cells and has lower expression in matured postmitotic neurons [2]. Importantly,* miR-9* regulates neurogenesis at the midbrain-hindbrain boundary in zebrafish brain models [3]. Another well-studied miRNA involved in brain development is* miR-124*.* miR-124* is recognized as a brain-specific miRNA and is the most abundant miRNA in the mouse brain [4].* miR-124* is expressed in the mature neurons of adult mouse brain and is upregulated in differentiating neurons [5]. miRNAs are also implicated in various neuropathologies [6], neurodegenerative diseases [7], and intellectual disabilities [8].

*miR-344* is a novel miRNA that was first reported in 2004 as one of the many miRNAs found in rat cortical neurons [9].* miR-344* is located on mouse chromosome 7, which contains 19 mature sequences [10]. A study by Royo et al. showed that* miR-344* was one of the imprinted small RNA genes at the Prader-Willi locus of the transgenic mouse model. It was predicted to map between* Ndn* and* Snrpn* genes located at the Prader-Willi domain on mouse chromosome 7 [11]. However,* miR-344* was not detected in homologous human Prader-Willi domain at 15q11q13 or any nonrodent genomes [11]. Therefore,* miR-344* is a nonconserved miRNA and it is specific to rodents.* miR-344* family had nine known isoforms,* miR-344a* to* miR-344i*. However, limited studies were carried out on a few of these isoforms and implicated with roles in various pathological disorders.* miR-344a* was found to be upregulated in the myocardium of lipopolysaccharide-treated rats. It was postulated that* miR-344a* was involved in endotoxin-induced myocardial injury [12].* miR-344h* was one of the miRNAs identified in a study that observed miRNA expressional alteration of a mouse hippocampus after a traumatic brain injury [13]. Another study also had showed that* miR-344b*,* miR-344d*, and* miR-344h* were downregulated in a neurotoxin-induced apoptosis in mouse MN9D cell line [14].

Studies revealed that* miR-344* is expressed during mouse brain development at E15.5 [10, 15] and in the adult mouse brain [16]. Recently,* miR-344-3p* was reported to be expressed in neural-specific regions during mouse embryonic development [10]. Although evidence had shown that* miR-344*, particularly* miR-344b* and* miR-344c*, was expressed in the developing mouse brain, the function of these miRNAs had yet to be ascertained. Besides the developing brain,* miR-344* had been implicated in mouse adipocyte differentiation [17, 18]. A high throughput microarray study revealed that* miR-344* was one of the 29 miRNAs identified which inhibits adipogenesis* via* Wnt signally pathway [18]. Subsequent study showed* miR-344* inhibited cell differentiation by targeting the Wnt/*β*-catenin signalling pathway [19]. Moreover,* miR-344* had been implicated in Huntington disease and acute respiratory distress syndrome animal models.* miR-344* was found downregulated in the brain of Huntington disease mouse models [20] while it was upregulated in the lungs of the rat model for acute respiratory distress syndrome [21].

In this study, we profiled the expression of* miR-344b *and* miR-344c* in mouse brain development via* in situ* hybridization at both embryonic and postnatal stages. A quantitative analysis was also carried out to determine the expression levels of these miRNAs in the central nervous system and multiple organs. Bioinformatics analysis was employed to predict the potential downstream target genes of* miR-344b* and* miR-344c*. A luciferase protein suppression assay was then performed as a downstream measurement of miRNA efficiency on a target sequence.

## 2. Materials and Methods

### 2.1. Animals and Embryos

C57BL/6 mice were used throughout the study. Mice were kept in a 12 h light/12 h dark cycle with access to unlimited food and water; they were also not pharmacologically treated. Mice were mated overnight with a ratio of one male to two female mice. Female mice were considered pregnant with the presence of vaginal plug and the gestation time was designated as embryonic day (E) 0.5. Pregnant mice were culled, and embryos were harvested at E11.5, E13.5, E15.5, E17.5, and postnatal day (P) 1. Adult mice were anaesthetized with isoflurane inhalation followed by cervical dislocation, and the brain and multiple organs were harvested. The number of mice (*n*) used in each group was seven, where *n* = 2 for* in situ* hybridization and *n* = 5 for real-time quantitative polymerase chain reaction (RT-qPCR). Animals in this study were used in accordance with the Animal Care and Use Committee, Universiti Putra Malaysia (UPM/FPSK/PADS/BR-UUH/00469).

### 2.2. Tissue Processing

Embryonic mouse brains at E11.5, E13.5, E15.5, E17.5, and P1 were harvested and fixed in 4% paraformaldehyde (PFA) for 24 h in a refrigerated shaker. As for adult mouse brains, mice were first perfused with 4% PFA via transcardiac perfusion following anesthetization with 0.05 mL/10 g body weight of sodium pentobarbital (Sigma) through intraperitoneal injection. Then, the thoracic cavity was exposed to reveal the heart. A 25-gauge needle was inserted at the apex of the heart, and the right atrium was lacerated with scissors. Mice were perfused with 1x phosphate-buffered saline and then with 4% PFA. After perfusion, mice were decapitated, and brains were harvested. The tissues were collected and fixed with 4% PFA with gentle agitation for 2 d at 4°C. The tissues were then subjected to standard tissue processing procedures (incubation with 10% formalin, 80% ethanol, 95% ethanol (twice), 100% ethanol (thrice), and xylene (thrice) for 1 h each, and incubation with paraffin wax (twice) for 2 h each) using a semiclosed bench top tissue processor (Leica TP1020). The tissues were then embedded in a paraffin block in a Tissue Embedding Station (Leica EG1160).

### 2.3. miRNA* In Situ* Hybridization

Briefly, paraffin sections of the brain (8 *µ*m) were deparaffinized, rehydrated, and fixed with PFA, followed by digestion with Proteinase K (1.2 *µ*g/*µ*L in 0.05 M Tris-HCl (pH 7.5) and 0.05 M EDTA). Then, sections were refixed with PFA and acetylated with 0.1 M triethanolamine, 0.178% (v/v) concentrated HCl, and 0.25% (v/v) acetic anhydride. Prehybridization was performed in a humidified chamber of 50% (v/v) formamide at 65°C as previously described [15]. Sections were covered with prehybridization buffer consisting of 50% (v/v) deionized formamide, 3x saline sodium citrate buffer, 1x Denhardt's solution, 1x phosphate-buffered saline, 1 mg/mL yeast total RNA, and 1 mg/mL Herring sperm DNA. After 2 h of prehybridization, custom-made* miR-344b*,* miR-344c*, or* miR-scrambled* locked nucleic acid probes (Exiqon) were added to the hybridization buffer to a final concentration of 0.020 pmol/*µ*L. Hybridization was carried out in an oven at 57°C for 16 h as per the manufacturer's recommendations.

After hybridization, sections were washed with serial concentrations of saline sodium citrate buffer (2x, 1x, 0.5x, and 0.1x) for 15 min each at 48°C. Then, they were rinsed with 0.1x saline sodium citrate and preblocking solution (0.1 M Tris-HCl (pH 7.5), 0.15 M NaCl, and 240 *µ*g/mL Levamisole (Sigma)) for 5 min each at room temperature. In a humidified chamber, sections were blocked with 5% (v/v) heat-inactivated fetal calf serum, 1% (v/v) blocking powder in maleate buffer, and 0.1% (v/v) Tween-20 for 1 h at room temperature. After blocking, sections were incubated with 0.00015 U (1 : 1000 dilution) of Fab fragments anti-Digoxigenin antibody conjugated with alkaline phosphatase (Roche Diagnostics) in blocking buffer for 1 h.

The sections were later incubated with alkaline phosphatase buffer (0.1 M Tris-HCl (pH 9.5), 0.1 M NaCl, 0.05 M MgCl_2_, 1% (v/v) Tween-20, and 240 *µ*g/mL Levamisole) for 10 min. After blocking, 0.06x nitro-blue tetrazolium/5-bromo-4-chloro-3-indolyl-phosphate, toluidine salt (NBT/BCIP) stock solution (Roche Diagnostics) in blocking buffer was added and incubated with sections for 5–8 d or until purple coloration sufficiently developed. Then, the sections were washed in Tris-EDTA buffer, pH 8.0 (0.01 M Tris-HCl (pH 7.5) and 0.001 M EDTA (pH 8.0)), for 10 min at room temperature and washed with 3 changes of fresh 1x PBS for 3 minutes at each step. For colocalization study, the sections were counterstained with eosin for 10 minutes at room temperature. The eosin stain was discarded and the slide was wiped dry. The sections were then dehydrated in a series of ethanol concentrations and xylene for 3 min each and subsequently mounted in DPX mounting medium and covered with a glass cover slip.

For immunofluorescence study, the sections were incubated in primary antibody, polyclonal rabbit anti-Tuj1 (Sigma-Aldrich), with dilution factor of 1 : 1000 in a humidified chamber at 4°C for 16 hours. Then, the sections were washed thrice with 1x PBS and incubated with secondary antibody, anti-rabbit AlexaFlour® 488 (ThermoFisher Scientific), with dilution factor of 1 : 1000 in a humidified chamber for four hours at room temperature. The antibody was discarded and the sections were rinsed thrice in 1x PBS and subsequently mounted in ProLong® Gold Antifade mounting media with DAPI (ThermoFisher Scientific) and covered with a coverslip. The slides were left to dry in the dark at room temperature before keeping in 4°C for storage.

### 2.4. RNA Isolation and RT-qPCR

Total RNA was isolated from C57BL/6 whole mouse brain and multiple organs (pancreas, thymus, skin, stomach, lung, spleen, liver, adipose tissue, ovary and fallopian tubes, testes, small intestine, heart, kidney, large intestine, and skeletal muscle) using TRIzol (Invitrogen) according to the manufacturer's protocol. A total of 2.0 *µ*g of total RNA was reverse transcribed into cDNA using 0.05 *µ*M of in-house designed stem-loop primer (5′-GTTGGCTCTG GTAGGATGCC GCTCTCAGGG CATCCTACCA GAGCCAAACA CWGTC-3′) with 2.5 *µ*M oligo (dT)_20_ (Invitrogen) and a SuperScript® III Reverse Transcriptase Kit (Invitrogen) with modifications to the manufacturer's protocol. The stem-loop primer was added after the initial denaturation step at 65°C, and cDNA was synthesized using stem-loop pulsed reverse transcription as previously described [15]. First strand cDNA contained a target site for universal reverse primer (5′-GTAGGATGCC GCTCTCAGG-3′) and Universal ProbeLibrary (UPL) probe #21 (Roche Diagnostics), which were used together with specific forward primers for* miR-344b* (5′-GGACCATTTA GCCAAAGCCT-3′) and* miR-344c* (5′-GCGTGATCTA GTCAAAGCCT-3′), respectively. Pre-PCR steps, qPCR steps, and subsequent analysis were performed using LightCycler® 480 software version 1.5 (Roche Diagnostics) [15]. Four-data point standard curves for all analyses were constructed based on equally pooled pre-PCR products.* U6* small nuclear RNA was used as a reference gene for normalization. Primers used to amplify U6 small nuclear RNA were 5′-CGCTTCGGCA GCACATATA-3′ (forward) and 5′-AAATATGGAA CGCTTCACGAAT-3′ (reverse).

### 2.5. Statistical Analysis

Five independent biological replicates were used in each experiment. qPCR results for* miR-344b* and* miR-344c* were normalized against the* U6* small nuclear RNA used as endogenous controls. One-way analysis of the variance was used to compare expression levels among the groups of samples. A *P* value of less than 0.05 was considered statistically significant.

### 2.6. *In Silico* Analysis

Four data mining tools, miRanda (August 2010 Release), miRDB (version 4.0, January 2012), TargetScan Mouse (Release 6.2, June 2012), and DIANA micro-T CDS (version 5.0), were used to identify the candidate target genes of* miR-344b* and* miR-344c*. Predicted downstream target genes were ranked according to the criteria set for each bioinformatics tool. Both conserved and nonconserved sites were used to cover a wide range of target sites across various species. Threshold values were set to determine the specificity and sensitivity of the prediction to identify the target genes using the four data mining tools. Using miRanda bioinformatics [22], the predicted data with good miRSVR score and nonconserved miRNA was downloaded. The top 40% of the predicted target genes were selected for further analysis. However, with miRDB [23, 24], target genes with a target score between 60 and 100 were selected for further analysis. In TargetScan Mouse [25], a minimum total context+ score of −0.12, irrespective of site conservation, was used in the analysis, and the top 40% of the selected genes were subjected to further analysis. On the other hand, target genes with a minimum miTG score threshold of 0.75 were selected for DIANA micro-T CDS [26, 27]. The predicted genes from all data mining tools were then compiled into a Venn diagram [28] to identify common target genes. Target genes that were predicted by at least three tools were subjected to expression analysis in Allen Brain Atlas [29]. Genes that were expressed in embryonic and adult stages were considered for the next downstream analysis. This set of predicted target genes was then subjected to the Protein Analysis through Evolutionary Relationships Classification System [30]. Genes that played a role in transcription or gene regulation were further streamlined and selected for validation.

### 2.7. Cell Culture, Transfection, and Luciferase Assay

HEK293 cells were cultured in Dulbecco's Modified Eagle's Medium (Sigma) supplemented with 10% fetal bovine serum, 1% L-glutamine, 1% nonessential amino acids, 1% sodium pyruvate, and 1% penicillin-streptomycin (all from GIBCO). Cells were plated in a 12-well plate and incubated at 37°C with 5% CO_2_ until they reached 90–95% confluence. Transfection of plasmids with* miR-344b*,* miR-344c*,* Olig2*, and* Otx2*, purchased from GeneCopoeia*™*, USA, was performed using Lipofectamine3000 (Invitrogen) as per the manufacturer's protocol. Each transfection experiment was performed in triplicate.

For the luciferase assay, HEK293 cells were cotransfected with 0.8 *µ*g of the pEZX-MT01 plasmid carrying the 3′UTR of the target gene and 0.8 *µ*g of the pEZX-MR04 plasmid carrying pre-miRNAs in six different transfection groups (Group 1: negative control luciferase plasmid + pre-miRNAs; Group 2: target gene + miR-scramble; Group 3: target gene; Group 4: negative control luciferase plasmid; Group 5: target gene + pre-miRNAs; Group 6: mock control). Negative control luciferase plasmid contained the firefly luciferase gene without 3′UTR, while ultrapure water was used as mock control. Firefly and Renilla luciferase were measured at 24, 36, 48, and 60 h after transfection using a Luc-Pair*™* miR Luciferase Assay kit (GeneCopoeia). The assay was read using a GLOMAX 96 Microplate Luminometer (Promega). Firefly luciferase was normalized against Renilla luciferase, which serves as a bioluminescence control.

## 3. Results

### 3.1. Spatiotemporal Expression Profiling of* miR-344b* and* miR-344c* during Mouse Brain Development

**Figure 1:**
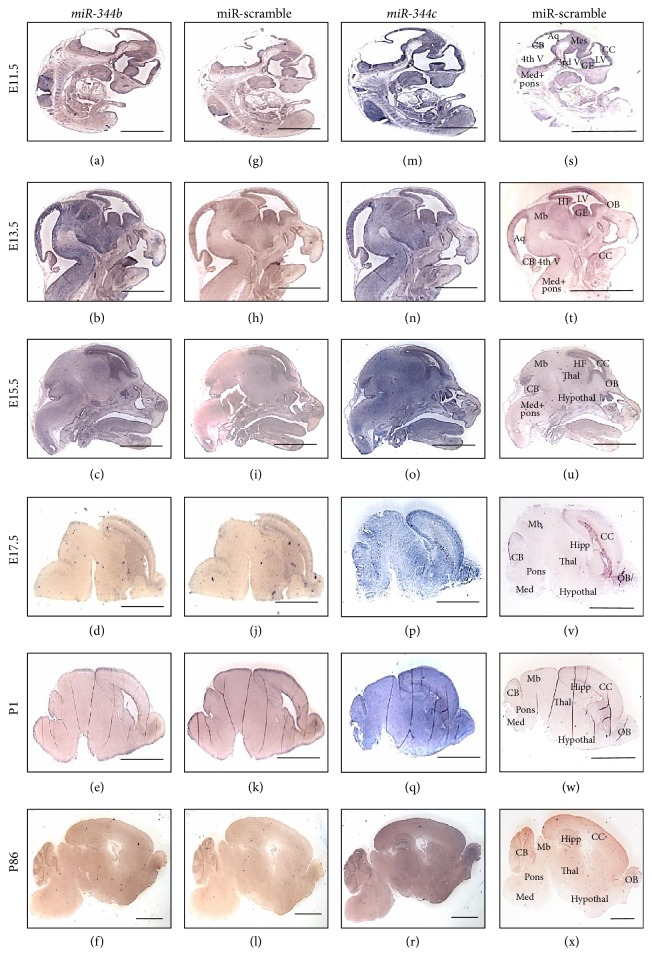
Spatiotemporal expression of* miR-344b* and* miR-344c* during mouse brain development. Sagittal brain sections showed spatial expression of* miR-344b* (a–f) with its miR-scramble (g–l), and* miR-344c* (m–r) with its miR-scramble (s–x). miR-scramble refers to negative control. Micrographs were taken at 1x magnification. Aq = aqueduct, CB = cerebellum, CC = cerebral cortex, GE = ganglionic eminence, HF = hippocampal formation, Hipp = hippocampus, Hypothal = hypothalamus, LV = lateral ventricle, Mb = midbrain, Med = medulla, Mes = mesencephalon, OB = olfactory bulb, Thal = thalamus, and V = ventricle. Scale bar, 3 mm.

To investigate the expression profiles of* miR-344b* and* miR-344c* during mouse brain development, we performed* in situ* hybridization on the sagittal plane of mice at E11.5, E13.5, E15.5, E17.5, P1, and P86 (*n* = 2).* miR-344b* was expressed throughout the entire embryonic brain at E11.5 (Figure 1(a)) and E13.5 (Figure 1(b)). At E15.5, stronger expression was observed in derivatives of the telencephalon (cerebral cortex and hippocampal formation) compared to other parts of the developing brain (Figure 1(c)). Subsequently, at E17.5, expression of* miR-344b* decreased and was not detectable from postnatal stages (Figures 1(d)–1(f)) onwards. On the other hand,* miR-344c* showed strong expression throughout the brain from E11.5 to P1 (Figures 1(m)–1(r)). In the adult stage (P86),* miR-344c* was not expressed in the brain with exception of the olfactory bulb and granular cell layer of the cerebellum (Figure 1(r)). miR-scramble was used on age-matched mouse brains as a negative control (Figures 1(g)–1(l) and 1(s)–1(x)).

**Figure 2:**
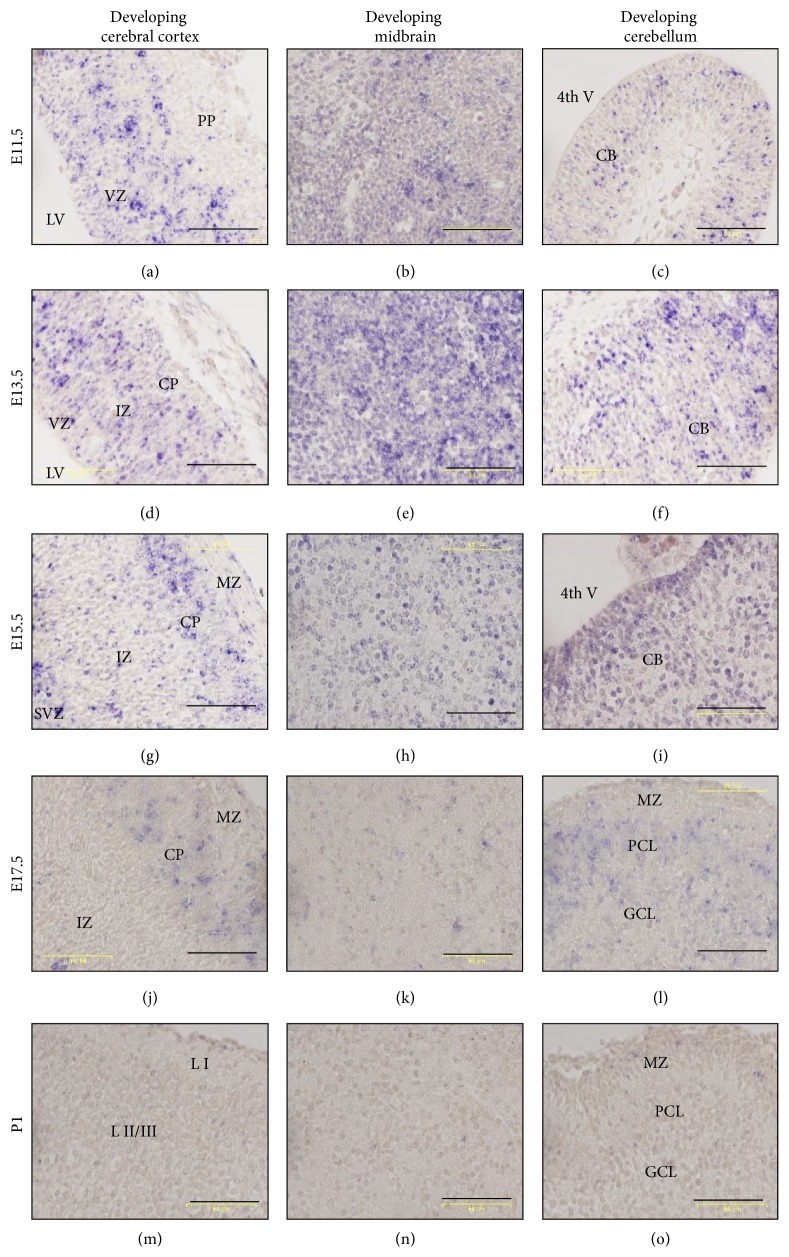
Expression of* miR-344b* in three developing brain regions: telencephalon (cerebral cortex), mesencephalon (midbrain), and rhombencephalon (cerebellum). Micrographs were taken at 40x magnification. miR-scramble showed no or minimal hybridization signals (Suppl. 2). 4th V = 4th ventricle, CB = cerebellum, CP = cortical plate, GCL = granular cell layer, IZ = intermediate zone, LI = layer I, L II/III = layer II/III, LV = lateral ventricle, MZ = molecular zone, PCL = Purkinje cell layer, PP = preplate, SVZ = subventricular zone, and VZ = ventricular zone. Scale bar, 64 *µ*m.

**Figure 3:**
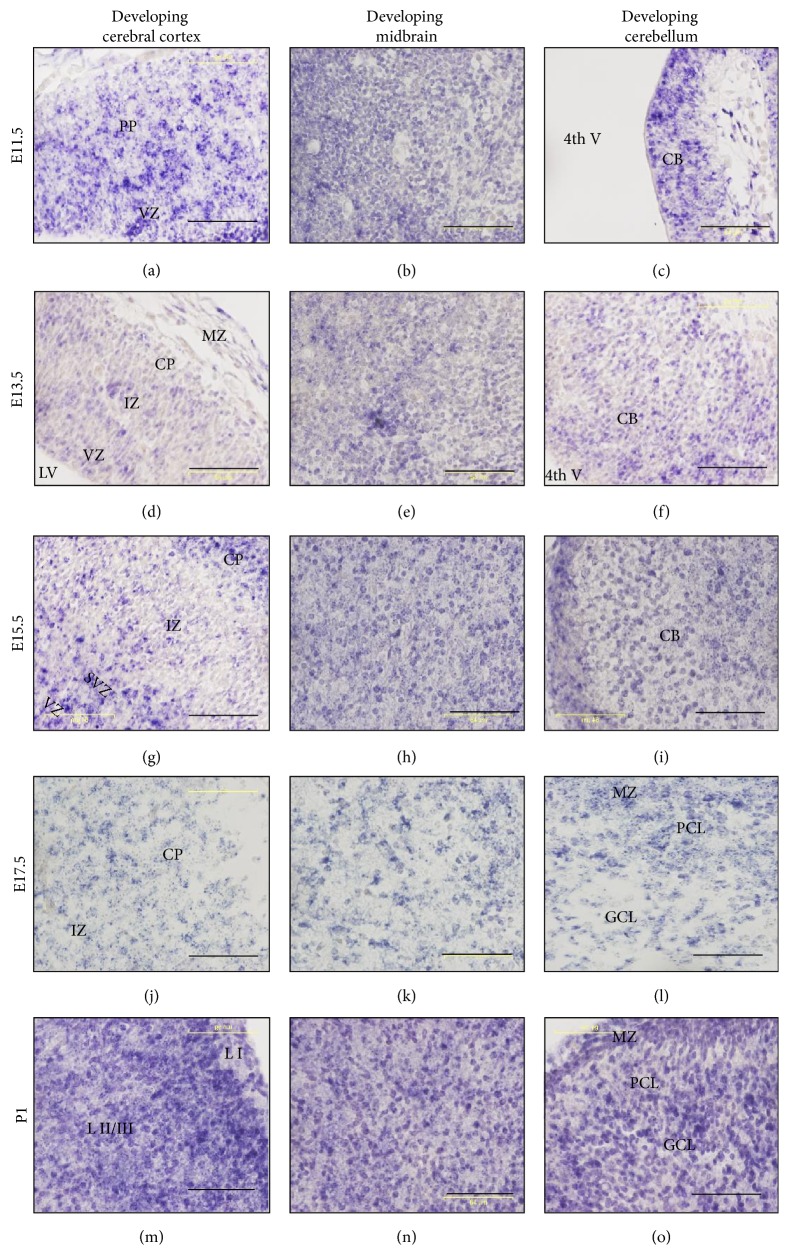
Expression of* miR-344c* in three developing brain regions: telencephalon (cerebral cortex), mesencephalon (midbrain), and rhombencephalon (cerebellum). Micrographs were taken at 40x magnification. miR-scramble showed no or minimal hybridization signals (Suppl. 3). 4th V = 4th ventricle, CB = cerebellum, CP = cortical plate, GCL = granular cell layer, IZ = intermediate zone, LI = layer I, L II/III = layer II/III, LV = lateral ventricle, MZ = molecular zone, PCL = Purkinje cell layer, PP = preplate, SVZ = subventricular zone, and VZ = ventricular zone. Scale bar, 64 *µ*m.

To further profile the spatial expression of* miR-344b* during brain development, sagittal sections of three primary areas of the brain, namely, the telencephalon (developing cerebral cortex), mesencephalon (developing midbrain), and metencephalon (developing cerebellum), were further evaluated (Figures 2 and 3). At E11.5,* miR-344b* expression was observed in the ventricular zone of the developing cerebral cortex (Figure 2(a)). At E13.5 and E15.5, its expression was found in the ventricular zone, intermediate zone, and cortical plate but not in the molecular zone of the cerebral cortex (Figures 2(d) and 2(g)). At E17.5,* miR-344b* was expressed exclusively in the cortical plate (Figure 2(j)). At P1,* miR-344b* was not detectable in any cortical layer of the cerebrum (Figure 2(m)) and continued to show no expression in the adult cerebral cortex.

In the mesencephalon,* miR-344b* was expressed throughout the developing midbrain at E11.5 (Figure 2(b)), E13.5 (Figure 2(e)), and E15.5 (Figure 2(h)). Expression of* miR-344b* was no longer detectable at late embryonic stages, E17.5 (Figure 2(k)), and postnatal stages P1 (Figure 2(n)) and P86. In the developing cerebellum,* miR-344b* was expressed in the cerebellar neuroepithelium at E11.5 (Figure 2(c)), E13.5 (Figure 2(f)), and E15.5 (Figure 2(i)). At E17.5,* miR-344b* was lowly expressed in the Purkinje and granular cell layer of the developing cerebellum (Figure 2(l)). As development progressed to P1,* miR-344b* was not expressed in any layer of the cerebellum (Figure 2(o)), nor was it expressed in the adult cerebellum.

In contrast to* miR-344b*,* miR-344c* was globally expressed throughout brain development from E11.5 to P1 and decreased in adulthood (Figures 1(g)–1(l)). At E11.5,* miR-344c* was expressed in both the ventricular zone and preplate of the developing cerebral cortex (Figure 3(a)). At E13.5 and E15.5, it continued to be expressed in the ventricular zone, intermediate zone, and cortical plate but not in the marginal zone of the developing cerebral cortex (Figures 3(d) and 3(g)). At E17.5,* miR-344c* continued to be expressed in the cortical plate and intermediate zone but not the marginal zone (Figure 3(j)). At P1,* miR-344c* was expressed in layers I, II, and III of the cerebral cortex (Figure 3(m)).

*miR-344c *was expressed throughout the developing midbrain from E11.5 to P1 (Figures 3(b), 3(e), 3(h), 3(k), and 3(n)) but not in the adult stage at P86. In the developing cerebellum,* miR-344c* was expressed in the cerebellar neuroepithelium at E11.5 (Figure 3(c)), E13.5 (Figure 3(f)), and E15.5 (Figure 3(i)). At E17.5 and P1,* miR-344c* was expressed in the molecular, Purkinje, and granular cell layers of the developing cerebellum (Figures 3(l) and 3(o)).

**Figure 4:**
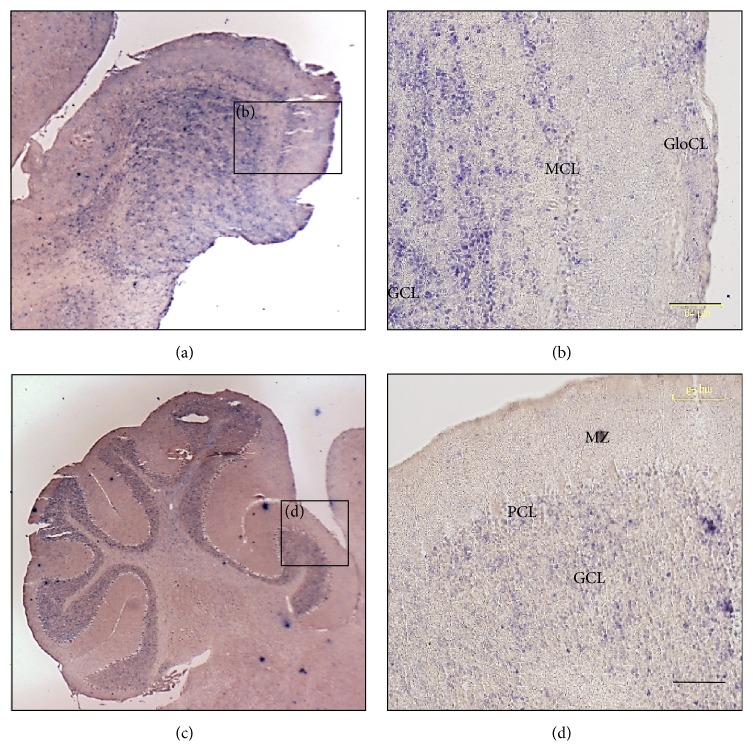
Expression of* miR-344c* in adult mouse brain. Sagittal sections of the olfactory bulb (a) and cerebellum (c) at 3x magnification. Insets (b) and (d) were taken at 20x magnification. GCL = granular cell layer, GloCL = glomerular cell layer, MCL = mitral cell layer, MZ = molecular zone, and PCL = Purkinje cell layer. Scale bar, 64 *µ*m.

Besides being expressed in adult (P86) cerebral cortex,* miR-344c* was also expressed in the adult olfactory bulb (Figure 4(a)). The miRNA was expressed in the molecular, mitral, and granular cell layers (Figure 4(b)). In the cerebellum (Figure 4(c)),* miR-344c* was lowly expressed in the granular cell layer (Figure 4(d)). In comparison,* miR-344b* was not expressed in both the adult mouse olfactory bulb and cerebellum.

### 3.2. Stem-Loop RT-qPCR Expression Analysis of* miR-344b *and* miR-344c*

**Figure 5:**
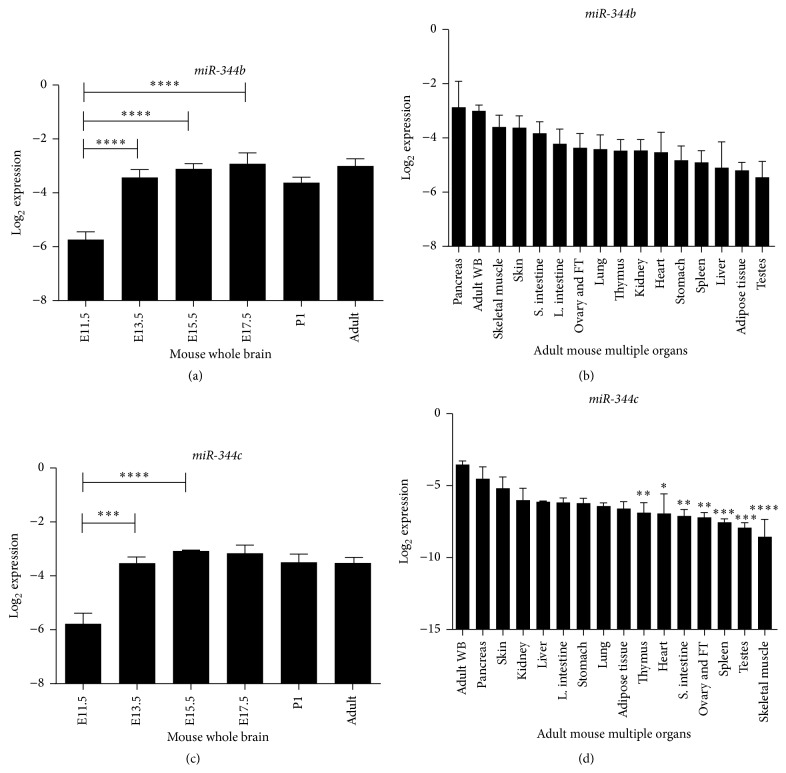
Expression profiles of* miR-344b* and* miR-344c* during mouse brain development and different adult mouse organs. Stem-loop RT-qPCR expression profiles of* miR-344b* and* miR-344c* during brain development (a, c) and in adult mouse multiple organs (b, d). In each analysis, the mean ± standard error of mean (SEM) for each tissue is presented in bar graphs. Log_2_ expression profiles of* miR-344b* are normalised to small nuclear RNA,* U6*. Asterisks denote the statistical significance at *P* < 0.05 (*∗*), *P* < 0.01 (*∗∗*), *P* < 0.001 (*∗∗∗*), and *P* < 0.0001 (*∗∗∗* *∗*) based on one-way ANOVA analysis. FT = fallopian tube, L. intestine = large intestine, S. intestine = small intestine, and WB = whole brain.

To quantify the expression of* miR-344b* and* miR-344c* (Figure 5), we performed stem-loop RT-qPCR in embryonic mouse whole brain and multiple organs of adult mice. Using whole brain samples (*n* = 5), a significant difference in* miR-344b* expression was found at E11.5, E13.5, E15.5, E17.5, P1, and adult brain samples (*P* < 0.0001; Figure 5(a)). Expression of* miR-344b* significantly increased from E11.5 to E13.5.* miR-344b* then continued to express until the adult stage (Figure 5(a)), which was in contrast with results from our* in situ* hybridization study. We further compared expression of* miR-344b* among various adult mouse organs and found no significant difference in its expression among them (*P* = 0.0609; Figure 5(b)). The adult mouse pancreas had the highest expression of* miR-344b*, followed by the brain, skeletal muscle, skin, small intestine, large intestine, ovary and fallopian tubes, lung, thymus, kidney, heart, stomach, spleen, liver, adipose tissue, and testes (Figure 5(b)).

The same analysis was performed on* miR-344c* using embryonic mouse whole brain and multiple adult mouse organs. Using whole brain samples (*n* = 5), a significant difference was observed in* miR-344c* expression at E11.5, E13.5, E15.5, E17.5, or P1, or in adult brain samples (*P* < 0.0001; Figure 5(c)).* miR-344c *was significantly increased from E11.5 to E13.5 and continued to express until the adult stage (Figure 5(c)). We further compared the expression of* miR-344c* in multiple adult organs and found significant differences in its expression among them (*P* < 0.0001; Figure 5(d)). The adult mouse brain had the highest* miR-344c* expression, followed by the pancreas, skin, kidney, liver, large intestine, stomach, lung, adipose tissue, thymus, heart, small intestine, ovary and fallopian tubes, spleen, testes, and skeletal muscle. It was also found that the expression of* miR-344c* in thymus, heart, small intestine, ovary and fallopian tubes, spleen, testes, and skeletal muscle was significantly lower compared to the brain (Figure 5(d)).

### 3.3. Predicted Target Genes of* miR-344b* and* miR-344c*

**Figure 6:**
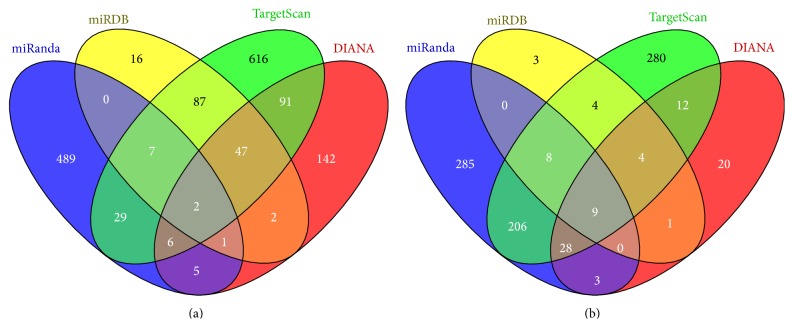
Venn diagram showing the predicted target genes of* miR-344b* and* miR-344c*. Targeted genes were predicted using miRanda, miRDB, TargetScanMouse, and DIANA micro-T CDS for* miR-344b* (a) and* miR-344c* (b). The total number of genes predicted by each software program is indicated in each circle.

To identify the genes targeted by* miR-344b *and* miR-344c*, we employed four online bioinformatics databases, namely, miRanda, miRDB, TargetScanMouse, and DIANA micro-T CDS. The Venn diagram summarized the number of genes targeted by* miR-344b* (Figure 6(a)) and* miR-344c* (Figure 6(b)) using the different databases. A total of 539 genes were predicted by miRanda, while 162 genes were predicted by miRDB. TargetScanMouse predicted 885 genes, and DIANA micro-T CDS predicted 302 genes. To increase the specificity of the predictions, we focused on genes predicted by three or four databases. Based on these criteria, a total of 63 target genes were predicted and seven genes were identified as transcription factors (*Jmjd1c*,* Med14*,* Olig2*,* Kbtbd7*,* Tox*,* St18*, and* Zranb2*). Of these genes, only four were expressed in both embryonic and adult mouse brain according to Allen Brain Atlas (*Jmjd1c*,* Olig2*,* Tox*, and* St18*).

As for* miR-344c*, 539 genes were predicted by miRanda, while 29 genes were predicted by miRDB. TargetScanMouse predicted 551 genes, and DIANA micro-T CDS predicted 85 genes. Nine genes were commonly identified by using a similar set of criteria (Figure 6(b)). These genes were* Otx2*,* Pnpla8*,* Erich1*,* Fam118a*,* Tmpo*,* Olfr1426*,* Pou4f1* (also known as* Brn3a*),* Tmem131*, and* Stau1*. Of these nine genes, only* Otx2* and* Pou4f1* were transcription factors expressed in both embryonic and adult mouse brains.

### 3.4. Target Gene Validation via Luciferase Assay

**Figure 7:**
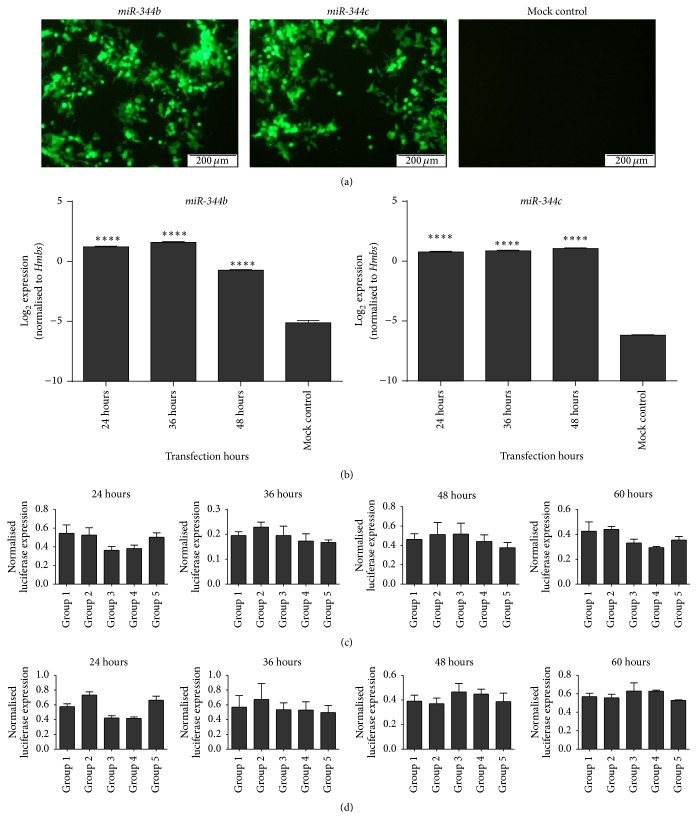
*Olig2* and* Otx2* expression was not suppressed by* miR-344b* and* miR-344c*. (a) Transfection efficiency of* miR-344b* and* miR-344c* expression vectors that contained eGFP reporter gene at 48 h. (b) Stem-loop RT-qPCR expression profiles of* miR-344b* and* miR-344c* in transfected HEK293 cells. Graph bars of each transfection period were represented as mean ± standard error of mean (SEM). Log_2_ expression profiles of* miR-344b* and* miR-344c* were normalized to* Hmbs*. Asterisks denote the statistical significance at *P* ≤ 0.0001 (*∗∗∗* *∗*) based on one-way analysis of variance. The luciferase assay was performed to validate the predicted target gene was a direct target of* miR-344b* (c) and* miR-344c* (d) 24, 36, 48, and 60 h after transfection. Group 1 = negative control luciferase vector + miRNA expression vector; Group 2 = target gene luciferase vector + miR-scramble; Group 3 = target gene luciferase vector; Group 4 = negative control luciferase vector; Group 5 = target gene luciferase vector + miRNA expression vector.

We chose* Olig2* and* Otx2* genes as potential targets of* miR-344b* and* miR-344c*, respectively, for further validation.* Olig2* has been established as a neuronal and glial fate determinant [31], and* Otx2* plays a role in formation and patterning of the rostral head [32]. We conducted a luciferase assay to determine whether* Olig2* and* Otx2* were targeted by* miR-344b* and* miR-344c*, respectively. The luciferase assay was performed 24, 36, 48, and 60 h (*n* = 3) after transfection in six different cotransfected groups to investigate expression of the miRNA and chimeric target gene over time. Transfection Groups 1 and 2 served as negative controls that determined the specificity of the miRNA to the target. Groups 3 and 4 were negative controls that determined the effect of host intrinsic factors on the chimeric targets. Group 5 was the assay test group, while Group 6 was a mock control for background luminescence. Fluorescence micrographs were captured to determine the transfection efficacy (Figure 7(a)). Using green fluorescent protein as an indicator, 70–80% of the cells were successfully transfected. Stem-loop RT-qPCR was carried out to determine the overexpression of* miR-344b* and* miR-344c* (Figure 7(b)). It showed that both miRNAs were present 24, 36, and 48 h after transfection after being normalized against* Hmbs*. Normalized luciferase bioluminescence was not downregulated at 24, 36, 48, or 60 h when the chimeric target gene* Olig2* was cotransfected with* miR-344b* (Figure 7(c)). The same experiment was performed for* miR-344c* and its chimeric target gene,* Otx2*. Similar to* miR-344b*, the normalized luciferase bioluminescence level was not affected by overexpression of* miR-344c* (Figure 7(d)).

### 3.5. Colocalization Study of* miR-344b* and* miR-344c*

**Figure 8:**
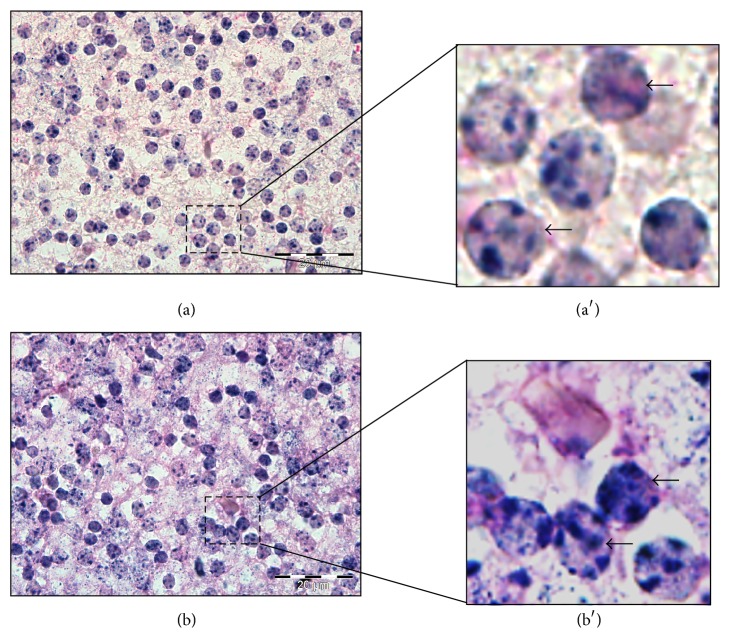
Localisation of* miR-344b* and* miR-344c* within a single cell. Sagittal sections of E15.5 midbrain showed that* miR-344b* (a) and* miR-344c* (b) were localised in the nucleus while the cytoplasm was counterstained with eosin. Insets (a′) and (b′) are enlarged field of (a) and (b), respectively. Arrow denotes the localisation of miRNAs within a single cell. Micrographs were taken at 100x magnification. Scale bar, 20 *µ*m.

Bioinformatics study had predicted* Olig2* and* Otx2* were target genes of* miR-344b* and* miR-344c*, respectively. However, these target genes were not targeted by their respective miRNAs as indicated in the luciferase assay. Therefore, we sought to determine the localization of* miR-344b* and* miR-344c* in the cells of the developing brain. Closer observations of ISH brain sections revealed that both* miR-344b* and* miR-344c* were localized to the nuclei instead of the cytoplasm of the cell (Figures 8(a) and 8(b)).

**Figure 9:**
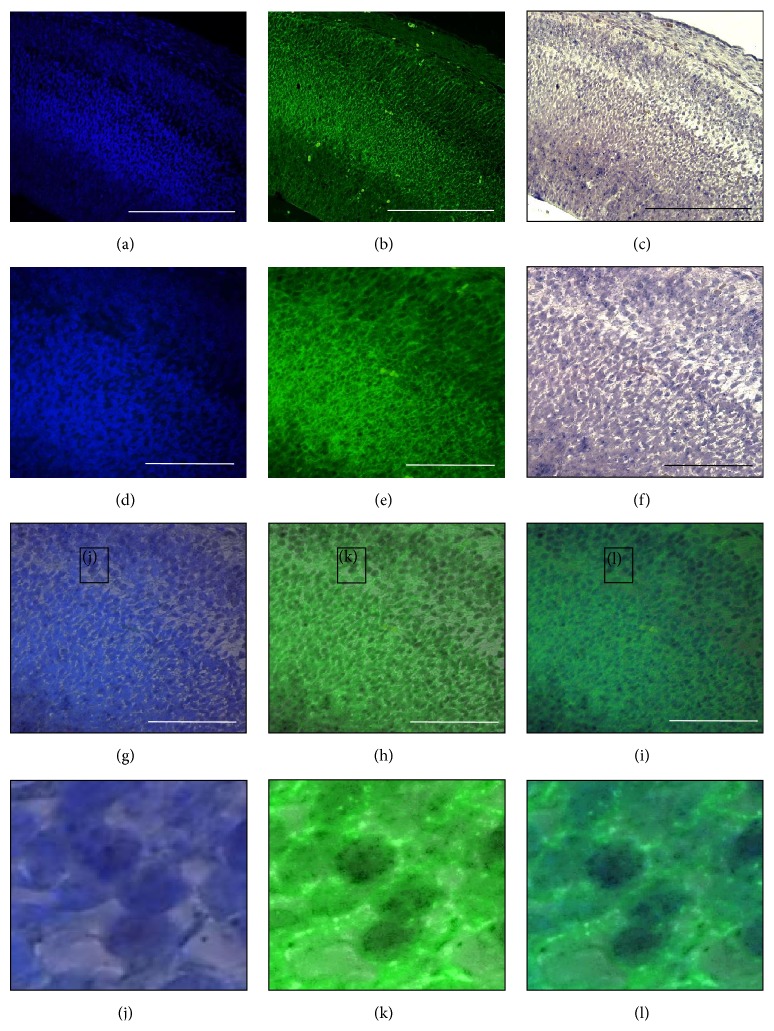
Expression of* miR-344b* colocalised with DAPI and Tuj1 immunofluorescence. Sagittal sections of E15.5 cerebral cortex with DAPI (a), Tuj1 (b), and brightfield (c) at 20x magnification. Higher magnification of DAPI (d), Tuj1, and brightfield (f) at 40x. Merge images of DAPI/brightfield (g), Tuj1/brightfield (h), and DAPI/Tuj1/brightfield (i). Insets (j), (k), and (l) are enlarged field of (g), (h), and (i), respectively. Scale bar at 20x, 100 *µ*m, while scale bar at 40x, 50 *µ*m.

**Figure 10:**
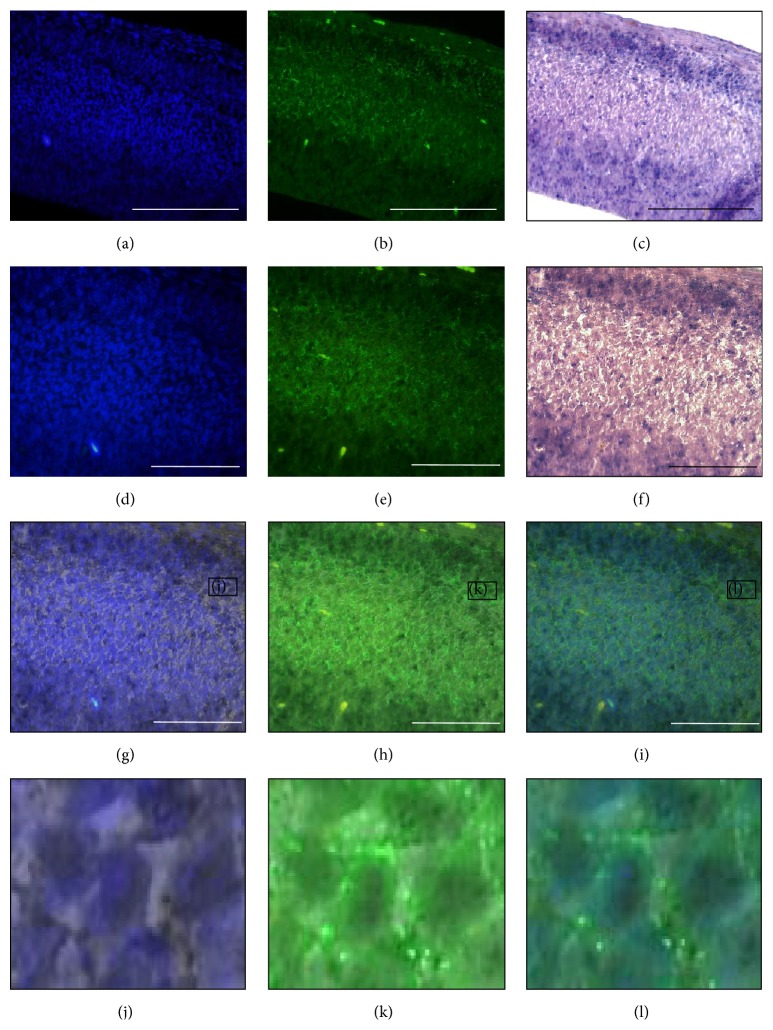
Expression of* miR-344c* colocalised with DAPI and Tuj1 immunofluorescence. Sagittal sections of E15.5 cerebral cortex with DAPI (a), Tuj1 (b), and brightfield (c) at 20x magnification. Higher magnification of DAPI (d), Tuj1, and brightfield (f) at 40x. Merge images of DAPI/brightfield (g), Tuj1/brightfield (h), and DAPI/Tuj1/brightfield (i). Insets (j), (k), and (l) are enlarged field of (g), (h), and (i), respectively. Scale bar at 20x, 100 *µ*m, while scale bar at 40x, 50 *µ*m.

Fluorescence microscopy was performed using UV channel for DAPI staining and FITC channel for Tuj1 staining. Sagittal sections of E15.5 mouse cerebral cortex were observed with DAPI (Figures 9(a), 9(d), 10(a), and 10(d)), Tuj1 (Figures 9(b), 9(e), 10(b), and 10(e)), and brightfield microscopy (Figures 9(c), 9(f), 10(c), and 10(f)). DAPI is known for staining the cell nucleus to distinguish individual cells. On the other hand, Tuj1 is a neuron-specific marker known for staining immature neuron. Both expressions of* miR-344b* and* miR-344c* were found coexpressed with DAPI (Figures 9(g), 9(j), 10(g), and 10(j)) as opposed to Tuj1 staining where green fluorescence was only found at the periphery of the cell (Figures 9(h), 9(k), 10(h), and 10(k)). Merged images of three different channels confirmed the locality of* miR-344b* and* miR-344c* in the nucleus of a neuronal cell (Figures 9(i), 9(l), 10(i), and 10(l)).

## 4. Discussion

In this study, we demonstrated comprehensive spatiotemporal expression of* miR-344b* and* miR-344c* throughout mouse brain development via* in situ* hybridization. The expression profiles for* miR-344b* and* miR-344c* were generally similar with slight differences at select brain regions or time points. Our findings concur with previous studies that showed* miR-344-3p* was expressed in embryonic and adult mouse brain [10]. Liu et al. performed time point* in situ* hybridization using whole embryos mounted at earlier embryonic stages (E9.5–E11.5) and whole brain sections at E15.5, E18.5, and adult stages. Our analysis provides further insight into* miR-344b* and* miR-344c* at other time points (E11.5, E13.5), as well as their expression in multiple adult mouse organs. Furthermore, a previous study by Ling et al. [15] showed two mature isoforms of* miR-344-3p* (*miR-344b* and* miR-344c*) were expressed in the whole developing mouse brain (E15.5) via Northern blot.

Many miRNAs have been found to be spatiotemporally expressed in the developing mouse brain.* Let-7*, one of the first miRNAs discovered, was found to be expressed in the neuroepithelium of E9.5 whole-mount mouse embryos, showing strong expression during neural differentiation processes [33].* miR-124* was shown to promote neurogenesis in the cerebral cortex [34] and regulate neurite growth during neuronal differentiation [35]. Moreover,* miR-9* is expressed in embryonic stem cells during neuronal differentiation [36]. A different review also suggested that overexpression of* miR-9* alters migration and proliferation processes of neural precursors [2]. The roles of spatiotemporally expressed* miR-344b* and* miR-344c* in brain development, however, are yet to be determined and warrant further characterization.

At higher magnifications,* miR-344c* was found globally expressed across the developing mouse brain. In contrast to* miR-344c*, expression of* miR-344b* was reduced at E17.5 and P1. In addition,* miR-344b* and* miR-344c* were expressed throughout the brain all sections, suggesting a wide regulatory role for these miRNAs during brain development, such as neuronal proliferation, migration, and differentiation. This expression pattern may suggest a possible role as housekeeping miRNA in maintaining basic cellular processes, as described elsewhere [37].

In the adult mouse brain (P86), only* miR-344c* was expressed and found localized to the olfactory bulb and subgranular zone of the cerebellum. Interestingly,* miR-344-3p* was also found to be primarily expressed in the olfactory bulb and cerebellar cortex of the adult mouse brain [10]. As* miR-344c* was expressed in the olfactory bulb, a brain region for odour recognition,* miR-344c* may be involved in transmission, integration, and processing of olfactory signals. In the cerebellum,* miR-344c* was found in the subgranular zone, which gives excitatory outputs to Purkinje cells.

Subsequent stem-loop RT-qPCR analysis of* miR-344b* and* miR-344c* was performed to validate our* in situ* hybridization findings. Both* miR-344b* and* miR-344c* had significantly increased from E11.5 to E13.5 and their expression remained in a steady state until adulthood. These findings were in contrast with our* in situ* hybridization results. Stem-loop RT-qPCR is a specific and sensitive approach to quantify individual miRNA present in the brain [38]. The expression of* miR-344b* and* miR-344c* may be widely diffused in the brain and it was not earlier detected via* in situ* hybridization, which was performed on a specific plane or section of the brain.

In addition to the whole mouse brain, we also performed similar analyses on* miR-344b* and* miR-344c* in various organs of the adult mouse. Comparison of multiple adult mouse organs showed the adult pancreas highly expressed both* miR-344b* and* miR-344c*.* miR-344b* was lowly expressed in adult mouse testes while skeletal muscles have the lowest expression of* miR-344c*. Besides the brain, other studies have shown that* miR-344* is expressed in the pancreas [39], lungs [21], and adipose tissue [18, 19], which concur with our findings in the current study. In contrast, miRNA array analysis suggested that* miR-344* was expressed specifically in the brain when compared to liver and heart tissues of the adult mouse [16].

Our study predicted* Olig2* and* Otx2* as the most probable targets of* miR-344b* and* miR-344c*, respectively. Both* Olig2* and* Otx2* are transcription factors that play a role in neurodevelopment. The prediction model was limited to transcription factors involved in the regulation of DNA transcription processes, which is the most common form of gene control [40]. It was used as a preliminary study to understand the functional role of* miR-344b* and* miR-344c*.* Olig2* has been reported to regulate mammalian brain development as a neuronal and glial cell determinant [41]. Furthermore,* Olig2* has antineurogenic properties and maintains multipotent neural progenitor cells [42]. As the predicted target of* miR-344c*,* Otx2* has been shown to be localized in the nuclei of cells of the olfactory bulb [43].* Otx2* is known to play a role in the formation and patterning of the developing brain [44].

Our subsequent attempt to validate* Olig2* and* Otx2* as downstream targets of* miR-344b* and* miR-344c* unexpectedly did not concur with our earlier predictions, as chimeric* Olig2* and* Otx2* were not suppressed by their respective miRNAs in the luciferase suppression assay. Both* miR-344b* and* miR-344c* were localized to the nucleus, suggesting that these mRNAs were not direct targets of these mature miRNAs. Although* Olig2* and* Otx2* were not validated as the targeted genes, other potential targets genes (Supplementary Tables 1 and 2, available online at http://dx.doi.org/10.1155/2016/1951250) predicted in the study warrant a more extensive validation in order to elucidate with the potential functional role of* miR-344b* and* miR-344c*.

A closer look into the expression profiles revealed that both the* miR-344b* and* miR-344c* were localized in the nuclei of neuronal cells, suggesting that they may function in nuclei rather than cytosol in a noncanonical manner. Higher magnification on these neuronal cells showed that* miR-344b* and* miR-344c* expressions were unevenly distributed in the nucleus, with an average of 5 foci per nucleus. These foci may be subnuclear structures known as paraspeckles [45]. Paraspeckles are the RNA-protein structures found in the interchromatin space of a mammalian cell. They are also the vital subnuclear domain to control gene expression by retaining nuclear mRNA. However, more studies are required to validate the potential role and mechanisms of* miR-344b* and* miR-344c* in the cell nucleus.

The noncanonical role of miRNAs has been described before, where mature miRNAs were transported back into the nucleus via importin-8 [46]. In the nucleus, these miRNAs may potentially complex with Argonaute proteins and bind primary miRNA transcripts. This prevents further downstream mechanisms related to miRNA biogenesis [47, 48]. In the absence of nuclear targets, the miRNAs are reexported into the cytoplasm in a process facilitated by exportin-1 [48].

Emerging evidence had suggested that nuclear miRNAs play a role in a noncanonical manner to regulate the biogenesis and function of other noncoding RNAs. A study by Tang et al. had shown that* miR-709* is localised in the mouse nucleus and binds to* miR-15a/16-1* recognition element and inhibits further processing of* miR-15a/16-1* primary transcript (pri-*miR-15a/16-1*) into* miR-15a/16-1* precursor (pre-*miR-15a/16-1*) [49]. A different study by Zisoulis et al. revealed that mature* let-7* was required in the association of protein Argonaute-like gene 1 (ALG-1) to* pri-let-7* transcripts in the nucleus of* Caenorhabditis elegans*. Disrupted ALG-1-pri-let-7 binding caused an increased* pri-let-7* in the nucleus but decreased mature* let-7* in cells [50]. These findings reinforced the hypotheses that miRNAs regulate the biogenesis of other miRNAs as well as its own.

## 5. Conclusion

In conclusion, our study shows that* miR-344b* and* miR-344c* are spatiotemporally expressed in the developing mouse brain. In multiple adult mouse organs, these novel miRNAs showed highest expression in the pancreas besides the brain. While* miR-344b* and* miR-344c* predicted downstream targets were* Olig2* and* Otx2*, respectively, these target genes were not validated via luciferase suppression assay. Further investigation to their expression pattern revealed that both miRNAs are expressed in the nucleus when counterstained with eosin. Immunofluorescence staining confirmed that* miR-344b* and* miR-344c* were expressed in the nucleus of the neurons.

## Supplementary Material

Supplementary materials contain two tables which described the list of commonly predicted target genes of *miR-344b* and *miR-344c* (Supplemental Table 1 and 2 respectively), one figure which showed the specificity of the stem-loop RT-qPCR assay (Supplemental Figure 1), and two figures which showed negative control staining (miR-scramble) for *miR-344b* and *miR-344c* (Supplemental figure 2 and 3 respectively).
