# Supplementary material for: Spatiotemporal Expression and Molecular Characterization of miR-344b and miR-344c in the Developing Mouse Brain
Source: Neural Plast. 2016 Mar 1;2016:1951250. doi: 10.1155/2016/1951250 (PMC4791505; doi:10.1155/2016/1951250)
Supplement: Supplementary file 1 — Supplementary materials contain two tables which described the list of commonly predicted target genes of miR-344b and miR-344c (Supplemental Table 1 and 2 respectively), one figure which showed the specificity of the stem-loop RT-qPCR assay (Supplemental Figure 1), and two figures which showed negative control staining (miR-scramble) for miR-344b and miR-344c (Supplemental figure 2 and 3 respectively). [file 1951250.f1.pdf]

Supplementary Figure Captions

Suppl. figure 1 Stem-loop pulsed reverse transcription (RT)-quantitative PCR assay validation confirms that the assay is specific to *miR-344b* and *miR-344c*. PCR was carried out for (A) *miR-344b* and (C) *miR-344c* respectively on 50ng, 100ng, 150ng, 200ng and 300ng of spiked genomic DNA (gDNA) to serve as contamination. Minus RT (- RT) control was used to amplify potential gDNA present during RNA extraction. E15.5 mouse brain was used as positive control, where a single band was observed. Random amplification was observed with various concentrations of gDNA contaminants on the 2% (w/v) TAE (Tris-acetic-EDTA) agarose gel. However, no amplification was detected on qPCR using UPL Probe #21 in (B) *miR-344b* and (D) *miR-344c* respectively.

Suppl. figure 2 The miR-scramble staining serve as a negative control for *miR-344b in situ* hybridization analysis involving three developing brain regions: telencephalon (cerebral cortex), mesencephalon (midbrain), and rhombencephalon (cerebellum). Micrographs were taken at 40x magnification. 4th V = 4th ventricle, CB = cerebellum, CP = cortical plate, GCL = granular cell layer, IZ = intermediate zone, LI = Layer I, L II/III = Layer II/III, LV = lateral ventricle, MZ = molecular zone, PCL = Purkinje cell layer, PP = preplate, SVZ = subventricular zone, VZ = ventricular zone. Scale bar, 64 µm.

Suppl. figure 3 The miR-scramble staining serve as a negative control for *miR-344c in situ* hybridization analysis involving three developing brain regions: telencephalon (cerebral cortex), mesencephalon (midbrain), and rhombencephalon (cerebellum). Micrographs were taken at 40x magnification. 4th V = 4th ventricle, CB = cerebellum, CP = cortical plate, GCL = granular cell layer, IZ = intermediate zone, LI = Layer I, L II/III = Layer II/III, LV = lateral ventricle, MZ = molecular zone, PCL = Purkinje cell layer, PP = preplate, SVZ = subventricular zone, VZ = ventricular zone. Scale bar, 64 µm.

Supplementary Figure 1

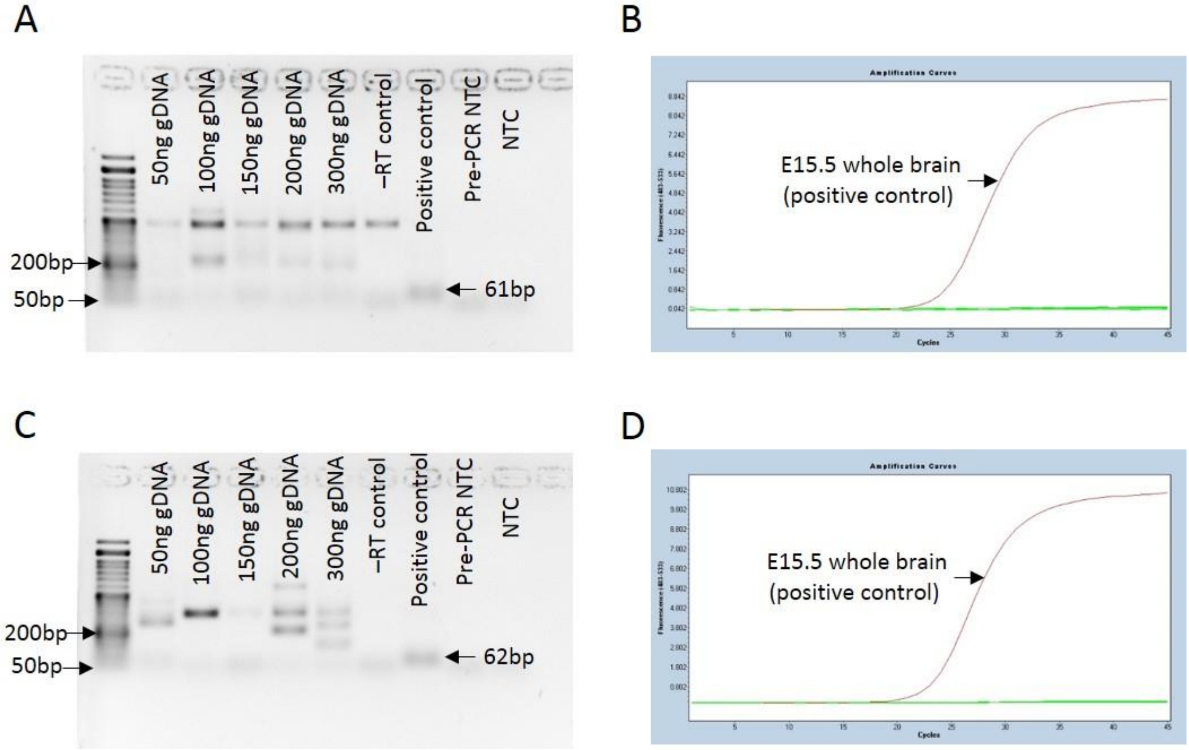

Supplementary Figure 2

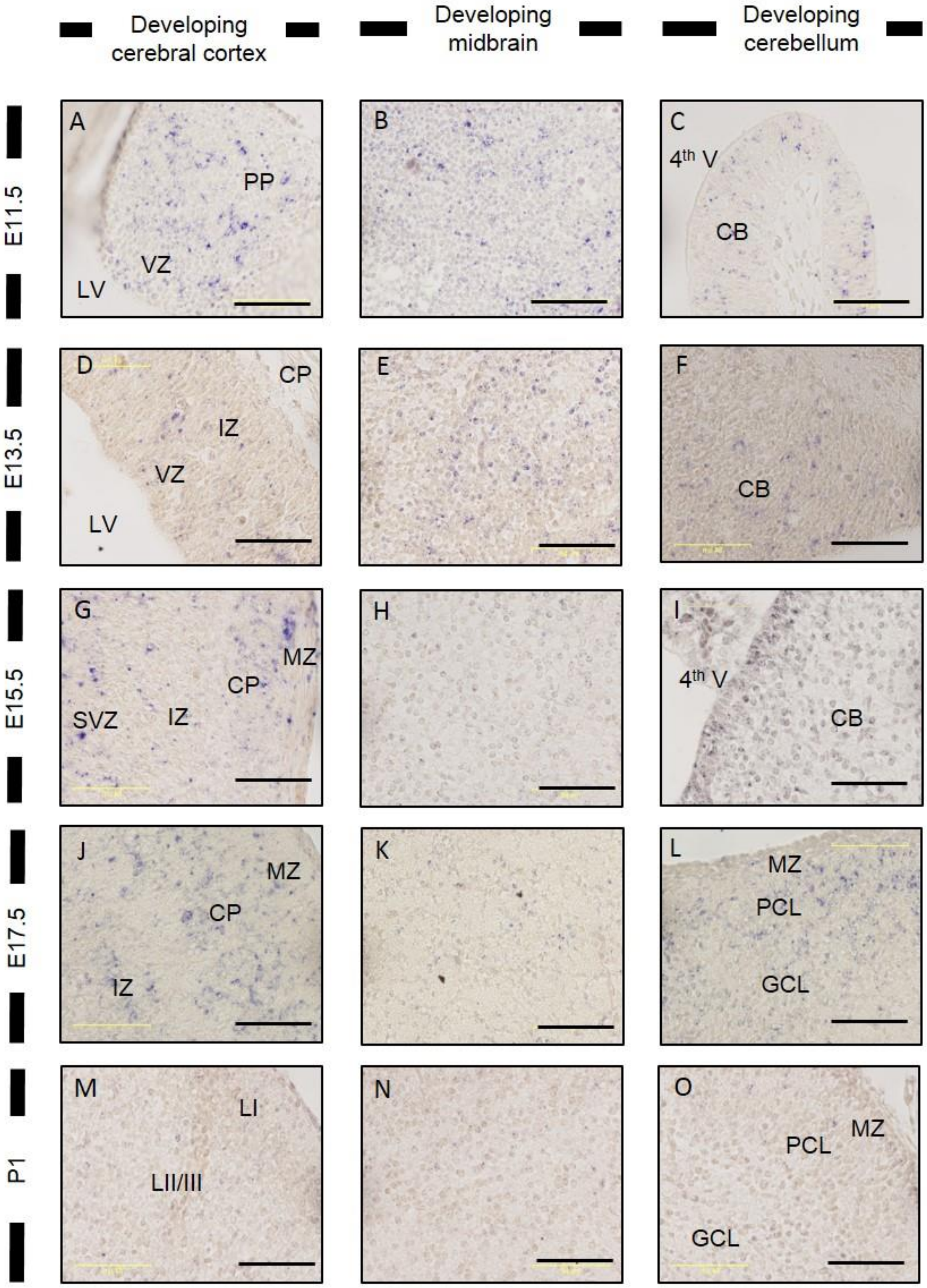

Supplementary Figure 3

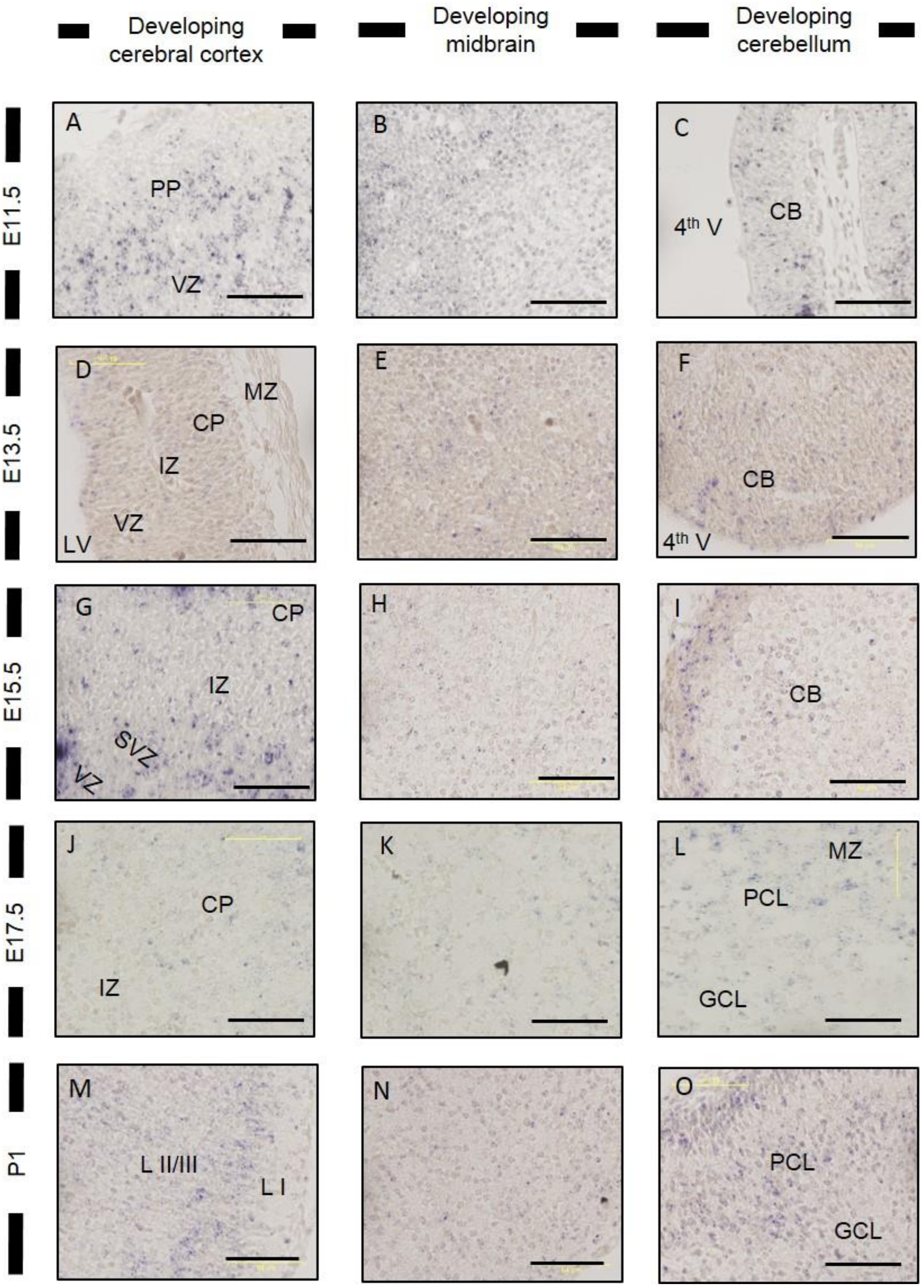

Supplementary Table 1

List of commonly predicted target genes of *miR-344b* by three or four bioinformatics software

Software	Predicted Target Genes			
**miRanda, miRDB, TargetScan, DIANA**	*Mtmr10*	*Dmd*		
**miRanda, miRDB, TargetScan**	*Unc80*	*Slc25a3*	*Isca1*	
	Gucy1a2	*Agtr2*	*Nmur2*	
	Gins3	*Mtmr10*	*Dmd*	
**miRDB, TargetScan, DIANA**	*Jmjd1c*	*Kbtbd7*	*Nol8*	*Socs6*
	Fgl2	*Erlin2*	*Dusp3*	*St18*
	Apc	*Phlpp1*	*Sh3bgrl*	*Ccdc80*
	Med14	*C130060K24Rik*	*Cndp1*	*A330021E22Rik*
	Urb2	*Cacnb4*	*Lrrc4c*	*Kitl*
	Actr2	*AU019823*	*D4Bwg0951e*	*1810037I17Rik*
	4930422G04Rik	*Ppip5k2*	*Srsf1*	*Gabra6*
	Tpp2	*Kif21a*	*Eea1*	*Gpr101*
	Ept1	*6030405A18Rik*	*Tox*	*Slc20a1*
	Olig2	*Fam151b*	*Lonrf3*	*A830018L16Rik*
	Sgpp1	*Tlk1*	*Ccdc14*	*Dse*
	Exo1	*Fgfr2*	*Ibsp*	*Mtmr10*
	Dmd			
**miRanda, TargetScan, DIANA**	*Fmr1*	*Atrx*	*Rab14*	*Zfp800*
	Ghr	*Gad2*	*Mtmr10*	*Dmd*
**miRanda, miRDB, DIANA**	*Zranb2*	*Mtmr10*	*Dmd*	

Note: Targeted genes highlighted in red were identified as transcription factors. Only *Jmjd1c*, *Olig2*, *Tox* and *St18* were expressed in both embryonic and adult mouse brain.

Supplementary Table 2

List of commonly predicted target genes of *miR-344c* by three or four bioinformatics software

Software	Predicted Target Genes			
**miRanda, miRDB, TargetScan, DIANA**	*Otx2*	*Fam118a*	*Olfr1426*	*Tmem131*
	Pnpla8	*Tmpo*	*Pou4f1*	*Stau1*
	Erich1			
**miRanda, miRDB, TargetScan**	*Adam9*	*Slco2b1*	*Acap2*	*Atxn1l*
	Plxnc1	*Gnptg*	*Kdm1a*	*Gda*
	Otx2	*Fam118a*	*Olfr1426*	*Tmem131*
	Pnpla8	*Tmpo*	*Pou4f1*	*Stau1*
	Erich1			
**miRDB, TargetScan, DIANA**	*Zfp959*	*Cetn1*	*Scrt2*	*Col6a4*
	Otx2	*Fam118a*	*Olfr1426*	*Tmem131*
	Pnpla8	*Tmpo*	*Pou4f1*	*Stau1*
	Erich1			
**miRanda, TargetScan, DIANA**	*Abcg1*	*Siah2*	*Rnf13*	*Manea*
	Emr4	*Fbxo11*	*Gm5460*	*Mmp25*
	Cks1b	*Esrrg*	*Fbln7*	*Tigd2*
	Gad1	*Mtmr10*	*Tyk2*	*Mapkapk3*
	Cbx4	*Gm4894*	*Itgb8*	*Fat4*
	Mex3c	*Adpgk*	*Arhgap24*	*Rpusd2*
	Mllt11	*Rad23b*	*Syngr2*	*2310047B19Rik*
	Otx2	*Fam118a*	*Olfr1426*	*Tmem131*
	Pnpla8	*Tmpo*	*Pou4f1*	*Stau1*
	Erich1			
**miRanda, miRDB, DIANA**	*Otx2*	*Fam118a*	*Olfr1426*	*Tmem131*
	Pnpla8	*Tmpo*	*Pou4f1*	*Stau1*
	Erich1			

Note: Targeted genes highlighted in red were identified as transcription factors. Only *Otx2* and *Pou4f1* were expressed in both embryonic and adult mouse brain.
